# Adjuvant Radiotherapy for Upper Tract Urothelial Carcinoma: Systematic Review and Meta-Analysis

**DOI:** 10.3390/curroncol30010002

**Published:** 2022-12-20

**Authors:** Osbert Zalay, Michael Yan, Samantha Sigurdson, Shawn Malone, Francisco Emilio Vera-Badillo, Aamer Mahmud

**Affiliations:** 1 Division of Radiation Oncology, University of Ottawa, Ottawa, ON K1H 8L6, Canada; 2 Department of Radiation Oncology, Princess Margaret Cancer Centre, Toronto, ON M5G 2C1, Canada; 3 Department of Oncology, McMaster University, Hamilton, ON L8V 5C2, Canada; 4 Department of Oncology, Queen’s University, Kingston, ON K7L 5P9, Canada; 5 Canadian Cancer Trials Group, Queen’s Cancer Research Institute, Kingston, ON K7L 2V5, Canada

**Keywords:** genitourinary, upper tract urothelial carcinoma, proximal ureter, renal pelvis, adjuvant radiotherapy, external beam, locoregional control, survival, systematic review, meta-analysis

## Abstract

Purpose: Upper tract urothelial carcinoma (UTUC) is a rare form of malignancy comprising only 5% of urothelial cancers. The mainstay of treatment is radical nephroureterectomy (RNU) with bladder cuff excision. Neoadjuvant or adjuvant chemotherapy is often used in locally advanced disease. The role of adjuvant radiotherapy (RT), however, remains controversial. To further explore the potential role of adjuvant RT, we performed a systematic review and meta-analysis of the literature from 1990 to present. Methods and Materials: We identified 810 candidate articles from database searches, of which 67 studies underwent full-text review, with final inclusion of 20 eligible studies. Among the included studies, there were no randomized controlled trials and a single prospective trial, with the remainder being retrospective series. We performed quantitative synthesis of the results by calculating the pooled odds ratios (OR) for the primary outcome of locoregional recurrence (LRR) and secondary outcomes of overall survival (OS), cancer-specific survival (CSS) and distant recurrence (DR). Results: Adjuvant RT, which was mostly prescribed for locally advanced or margin-positive disease following RNU, significantly reduced locoregional recurrence risk OR 0.43 (95% CI: 0.23–0.70), and the effect remained significant even following subgroup analysis to account for adjuvant systemic therapy. The effect of adjuvant RT on 3-year OS, 5-year CSS and DR was non-significant. However, 5-year OS was unfavourable in the adjuvant RT arm, but study heterogeneity was high, and analysis of small-study effects and subgroups suggested bias in reporting of outcomes. Conclusions: Adjuvant RT in the setting of locally advanced UTUC improves locoregional control following definitive surgery, but does not appear to improve OS. Higher-quality studies, ideally randomized controlled trials, are needed to further quantify its benefit in this setting, and to explore multi-modal treatments that include systemic agents given concomitantly or sequentially with RT, which may offer an OS benefit in addition to the locoregional control benefit of RT.

## 1. Introduction

Upper tract urothelial carcinoma (UTUC) -is rare, consisting of only 5% of urothelial malignancies and 10% of renal neoplasms [1,2,3]. The annualized incidence of UTUC in Western countries is estimated to be around 2 cases per 100,000 person-years [4]. UTUC is associated with a high risk of both local and distant recurrence [1,5,6], with prognosis being strongly dependent on the stage of disease at time of presentation [4,7,8,9]. Smoking and exposure to aristolochic acid (present in some Asian herbal medicines) are the two most common risk factors for development of UTUC [10,11,12]. Other epidemiologic factors associated with incidence of UTUC include gender, geographic location and occupation [13]. Incidence of UTUC in men is approximately two times higher than in women, and some studies report gender-specific differences in outcomes, although the evidence is not conclusive [4,5,14,15]. Incidence is also higher in some areas of Europe such as the Bas-Rhin region of France and the Balkan states, as well as parts of Asia. In terms of occupation, sailors, print workers and welders having the highest standardized incidence ratio amongst various occupations, but smoking is likely a confounder [13]. An association with Lynch syndrome and mismatch repair genes has also been identified [16].

UTUC behaves differently from lower urothelial tract cancers, likely due to different embryonic, anatomical and molecular factors that also influence the response to therapeutic modalities such as chemotherapy and radiation [17,18]. One study looking at mutations in UTUC identified *TP53*, *PIK3CA*, and *FGFR3* mutations as possible driver mutations, differing from urothelial carcinoma of the bladder; as well, patients with UTUC had lower PD-L1 expression than those with bladder cancer [19]. Expression of extracellular matrix proteins likewise differs in the upper urothelial tract from the urinary bladder, and the embryologic origin of the renal pelvis and ureter from the mesonephric duct means the tissues are susceptible to microsatellite instability, which provides a rationale for the association between UTUC and Lynch syndrome [16,17,18].

The primary modality for curative management of UTUC is surgical, namely radical nephroureterectomy [5]. The role of peri-operative treatments is less clear, although recent meta-analyses have demonstrated survival benefit for peri-operative chemotherapy in locally advanced disease [20,21]. (Muscle-invasive and non-organ-confined disease constitutes upwards of 50–60% of cases at time of clinical presentation [4]). In lower urothelial tract cancers, the role of radiotherapy is still evolving but accepted indications include concurrent chemoradiation for bladder preservation in muscle-invasive disease [22,23,24,25,26]. There is mounting evidence to suggest decreased recurrence risk with post-operative radiotherapy for locally advanced, high-grade or margin positive bladder cancer [27,28]. So far, however, there have been insufficient data to clearly define a role for adjuvant RT in the setting of locally advanced upper urothelial tract carcinoma. Previous analyses have shown mixed or even unfavourable outcomes with radiotherapy, with some groups reporting worse disease-specific and overall survival with the addition of adjuvant radiation [29,30]. However, much of the data are derived from smaller, older studies that utilized antiquated radiotherapy techniques.

There are emerging data supporting improved locoregional control with adjuvant radiation, primarily coming from institutions in Asia where the prevalence of UTUC and the use of post-operative radiotherapy for UTUC are more common [31,32,33,34]. However, the role of radiotherapy in this setting remains controversial, and is not currently standard of care [35]. Therefore, to help elucidate the potential role of adjuvant radiotherapy in urothelial carcinoma of the upper ureter or renal pelvis, we performed a systematic review and meta-analysis of the existing evidence to date.

## 2. Methods and Materials

### 2.1. Systematic Review of Literature

This study was conducted according to the Preferred Reporting Items for Systematic Reviews and Meta-Analyses (PRISMA) (Unique registry number: reviewregistry1491; https://www.researchregistry.com/browse-the-registry#registryofsystematicreviewsmeta-analyses/, accessed on 6 December 2022) [36]. Medline, PubMed, Embase, Cochrane and Google Scholar databases were queried. Search results were imported into Covidence (Veritas Health Innovation, Melbourne, Australia) for screening and eligibility assessment by two independent reviewers. The review looked at studies authored between 1990 to present. Non-English language articles were translated using automated translation software, including one article in Spanish and the other in Japanese. Both of these articles were peer-reviewed papers from respective national urological journals [37,38]. We included older studies and foreign-language publications to be as inclusive as possible given the rare nature of UTUC, and because of the geographic variation in UTUC incidence and treatment paradigms. Furthermore, by including older articles upon which current UTUC management guidelines are based, it is possible to compare and contrast the older studies with newer studies to determine the impact of more modern treatment modalities and to identify historic biases in study protocols.

Inclusion and exclusion criteria are listed in Table 1. Studies were included if they had transitional cell carcinoma (TCC) histology, ureter or renal pelvis for tumour location, muscle-invasive or locally advanced disease at time of diagnosis, and the use of post-operative external beam radiotherapy. Single case reports, review articles, expert opinion, or practice guidelines were excluded from the analysis. Studies were also excluded if they had pediatric patients, primarily non-TCC histology, dealt solely with recurrent or distant metastatic disease, distal urothelial cancers (bladder or urethra), or where patients did not receive adjuvant external beam radiotherapy. However, we still included single-arm studies in the systematic review of the literature, even though they were not included in the quantitative synthesis of data, as their reported radiotherapy outcomes for UTUC were still informative [34,39,40].

### 2.2. Data Extraction

Baseline factors included study type, institution(s), number of patients, median patient age, gender ratio (M:F), tumor location, tumor T stage, nodal status, type of surgery (e.g., radical nephroureterectomy vs. partial resection), surgical margin status, the number of patients receiving chemotherapy, the number receiving adjuvant radiotherapy, and the radiation dose-fractionation schedules. Our primary outcome of interest was locoregional recurrence (LRR). Secondary outcomes analysed included 3- and 5-year overall survival (OS), 3- and 5-year cancer-specific survival (CSS), progression-free survival, and distant recurrence (DR). We compared outcomes of patients treated with adjuvant radiotherapy following radical nephroureterectomy or partial resection versus those treated without adjuvant radiotherapy. However, the comparator arm was broadly defined to include either no therapy following surgery, chemotherapy alone, or salvage radiotherapy, as this differed between studies.

### 2.3. Quality Assurance

Study quality was evaluated using the validated revised methodological index for non-randomized studies (MINORS) criteria [41], which assesses 12 quality indices for comparative studies and 8 for non-comparative studies, respectively. The common indices pertain to the following: (i) study aim; (ii) inclusion criteria; (iii) prospective data collection; (iv) study endpoints; (v) unbiased assessment; (e.g., blinding) (vi) appropriateness of follow-up period; (vii) loss to follow-up; and (viii) prospective calculation of study size. The additional 4 indices for comparative studies include: (ix) adequate control/comparator group (x) contemporary groups (i.e., non-historical comparison) (xi) baseline equivalence (e.g., group similarity and confounders); and (xii) adequacy of statistical analyses. Each index is assigned a score of 2 for reported/adequate, 1 for reported/not-adequate and 0 for unreported or absent. The ideal cumulative score for comparative studies is 24, and for non-comparative studies or case series it is 16.

### 2.4. Statistical Analysis

All statistical analyses were performed in MATLAB (The Mathworks, Natick, MA, USA). Studies that had only a single-arm with respect to radiotherapy (e.g., case series) were included for review but did not contribute to the meta-analysis. Individual odds ratios for each study pertaining to a given outcome were calculated, and within-study variance was estimated by Woolf’s method [42]. If the odds ratio of a given study was undefined, due to sparse data resulting in cell counts of zero, a continuity correction of adding 0.5 to all cell counts for that study was applied [43]. Pooled odds ratios (*ORp*) were computed for both fixed and random effects models using Mantel-Haenszel and DerSimonian-Laird estimators, respectively [44,45]. Although the fixed and random effects pooled estimates are provided in the forest plots for each outcome, only the random effects model *ORp* is reported in the text.

Because the primary outcome of locoregional control for adjuvant radiotherapy includes studies where adjuvant chemotherapy was administered, either concomitantly or sequentially in relation to radiotherapy, a subgroup analysis was also performed to determine whether there was an effect of radiotherapy alone in the adjuvant setting. This was accomplished by examining locoregional recurrence rates in studies grouped by whether or not any of the patients received systemic chemotherapy in addition to radiotherapy, and the pooled odds ratios calculated for each subgroup and compared. Additional subgroup analyses were carried out to look at the effect of publication date, radiotherapy dose and study type on reported outcomes.

Study heterogeneity was assessed by computing the *Q*-statistic (weighted sum of squared difference of individual study odds ratios with the pooled estimate, which follows a chi-squared distribution with *n*-1 degrees of freedom), and *I*^2^ inconsistency measure deriving from the *Q*-statistic, expressed as a percentage [46]. For subgroup analysis, the difference between subgroups was estimated by pooling subgroup effects and quantifying the between-subgroup heterogeneity. Additional study bias was assessed for outcome measures that had pooled results which were statistically significant and of low heterogeneity, using Harbord’s Test, which is a modification of Egger’s test for small-studies effects that is more specific to binary outcomes when the odds ratio is the effect measure [47]. Results of Harbord’s Test were summarized in a radial plot, which regresses standardized effect size against the inverse standard error (the higher the inverse error, the lower the study variance). Small studies tend to have high variance, and thus small inverse standard error, and appear on the left side of the plot. Studies that are unbiased appear equally distributed above and below the |*Y*| = |*X*| line. A bias is present when the *Y*-intercept is significantly different from zero.

## 3. Results

### 3.1. Baseline Characteristics

**Figure 1:**
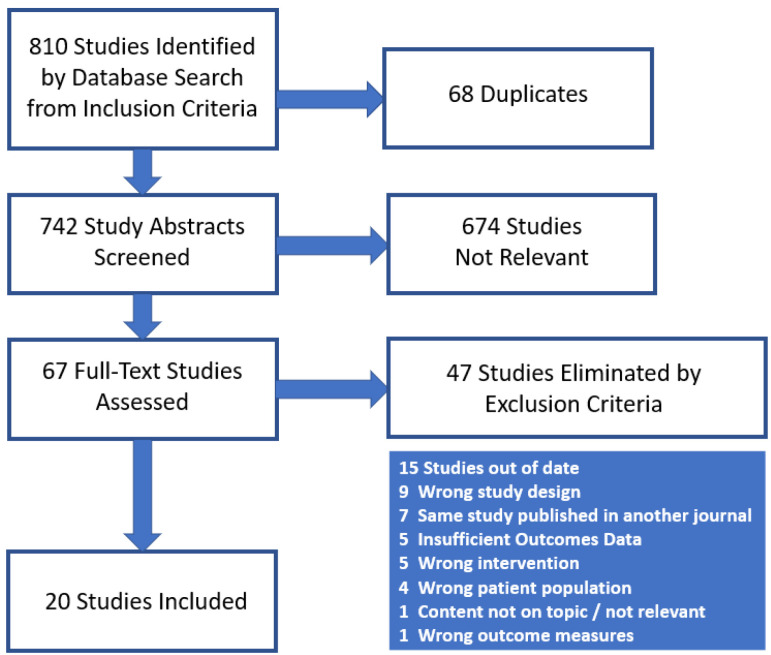
PRISMA flow chart for study selection.

We identified 20 studies eligible for inclusion in our review and meta-analysis (Figure 1), all of which were retrospective observational cohort studies with the exception of one prospective cohort study. Baseline data are listed in Table 2. Study dates ranged from 1991 to 2020. Four studies were multi-institutional, and two were based on analysis of the SEER (Surveillance, Epidemiology and End Results) database. The remainder were single-institution studies. The studies comprised a total of 6529 patients, of which 879 (13.5%) received post-operative radiotherapy in the adjuvant setting. The median pooled age of study participants weighted by study size was 71.9 years (range 61 to 74 years). The pooled gender ratio (M:F) was 1.4 (range 0.90 to 3.86). There were no statistically significant differences in the gender ratio between patients receiving adjuvant RT and those that did not [30,31,32,39,48,49,50]. Smoking status was only reported in one study [51]. Of the studies reporting specific clinical staging information (*n* = 16), 1914 out of 3834 patients (49.9%) had organ-confined T stage (T1 or T2), 1920 (50.1%) had locally advanced T stage (T3 or T4), and 399 out of 3362 (11.8%) had lymph node involvement of disease at time of treatment. 149 out of 1354 surgical patients (11.0%) had a positive margin from the studies that reported margin status (*n* = 10). 401 patients (6.1%) were recorded as having received chemotherapy (adjuvant, concomitant or salvage), although not all studies were clear on documenting whether patients had received chemotherapy. Those who received adjuvant radiotherapy primarily had locoregional or non-organ-confined disease (T3–4N0, T1–4N+), or positive margin status. The target volumes in most cases included the tumor bed (renal fossa and/or ureteric bed), sometimes with partial or full coverage of the bladder, especially if there was disease in the distal ureter. Elective nodal volumes, if treated, included the regional lymph nodes such as para-aortic and para-caval nodes. The majority of studies used 3D conformal technique for external beam radiotherapy, with standard fractionation (1.8–2 Gy per fraction), with some studies offering a 5–10 Gy boost for positive margins or gross residual disease. The pooled median treatment dose across studies reporting radiotherapy dose was 47.8 Gy (range 35–55 Gy), although the range of doses among patients from these studies spanned from as low as 20 Gy to as high as 66.6 Gy.

### 3.2. Study Quality

Table 3 summarizes the results of quality assurance for the 20 extracted studies using the MINORS criteria [41]. The majority of comparative studies were either in the moderate or higher quality range of scores, with a pooled comparative study score of 13.4. The 3 non-comparative studies (which were not used in the meta-analysis) had a composite score of 8.0. Overall, the studies demonstrate a moderate risk of bias based on these results.

### 3.3. Outcomes and Meta-Analysis

The outcomes extracted from the 20 included studies are summarized in Table 4, and include the primary outcome of locoregional recurrence, and secondary outcomes of overall survival, cancer-specific survival and distant recurrence.

#### 3.3.1. Locoregional Recurrence

**Figure 2:**
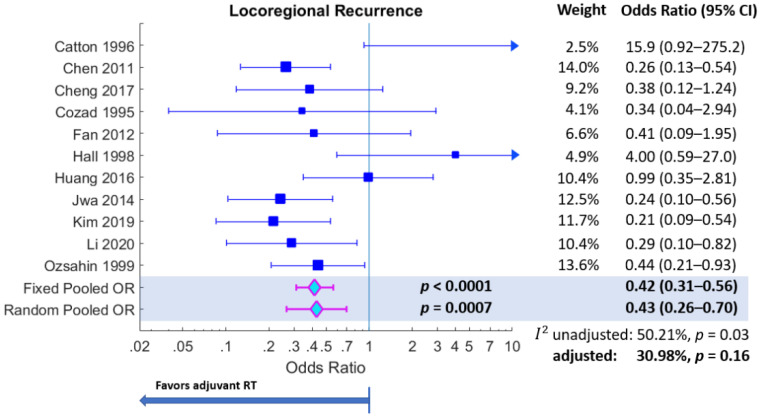
Forest plot for primary outcome of locoregional recurrence. Weights are from the random-effects model. The adjusted inconsistency measure (*I*^2^) pertains to recalculation of the measure after excluding the study by Catton et al. (1996).

Adjuvant radiotherapy reduced the odds of locoregional recurrence (Figure 2), with a pooled odds ratio of *ORp* = 0.43 (95% CI: 0.26–0.70), *p* = 0.007. Study heterogeneity was moderately high (*I*^2^ = 50.21%, *p* = 0.03). However, after excluding the study by Catton et al. (1996) from the analysis, because of the study’s disproportionate effect size impacting heterogeneity (more than 3.3 standard deviations from the norm, on account of sparse data with no recurrences in the comparator arm), the heterogeneity became non-significant (adjusted *I*^2^ = 30.98%, *p* = 0.16); the pooled odds ratio also improved in a favour of adjuvant RT (adjusted *ORp* = 0.38 (95% CI: 0.25–0.56), *p* < 0.0001).

#### 3.3.2. Overall Survival

**Figure 3:**
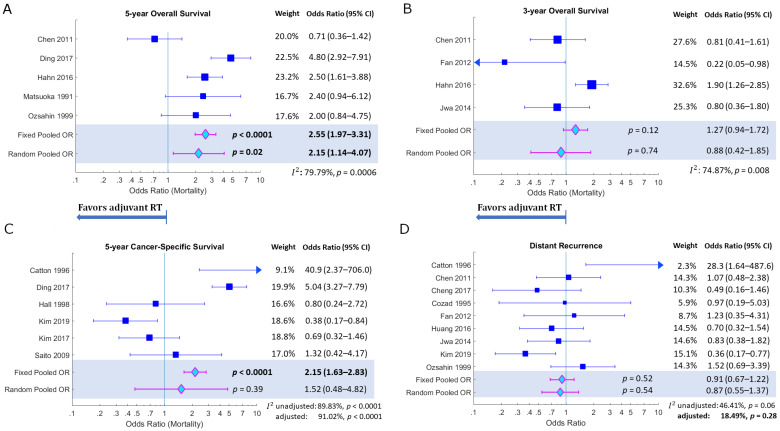
Forest plots for secondary outcomes of (**A**) 5-year overall survival; (**B**) 3-year overall survival; (**C**) 5-year Cancer-Specific Survival; and (**D**) Distant Recurrence.

5-year overall survival showed an unfavourable pooled odds ratio (Figure 3A), with higher chance of mortality at 5 years in the adjuvant radiotherapy arm (*ORp* = 2.15 (95% CI: 1.14–4.07), *p* = 0.02). However, study heterogeneity reporting 5-year overall survival was high (*I*^2^ = 79.79%, *p* = 0.0006). 3-year overall survival was not significantly different between the two arms (*ORp* = 0.88 (95% CI: 0.42–1.85), *p* = 0.74; *I*^2^ = 74.87%, *p* = 0.008) (Figure 3B).

#### 3.3.3. Cancer-Specific Survival

5-year cancer-specific survival was not significantly different between patients who received adjuvant radiotherapy and those who did not (Figure 3C) (*ORp* = 1.52 (95% CI: 0.48–4.82), *p* = 0.39). However, study heterogeneity was high (*I*^2^ = 89.83%, *p* < 0.0001). Two studies reported 2- or 3-year CSS [51,53], which slightly favoured the adjuvant radiotherapy arm (see Table 4), although the results were not statistically significant.

#### 3.3.4. Distant Recurrence

There was no discernible difference in distant metastatic recurrence between the two treatment arms, with most of the 9 studies reporting DR having odds ratios clustered about the null-effect line (Figure 3D). The pooled odds ratio was insignificant (*ORp* = 0.87 (95% CI: 0.55–1.37), *p* = 0.54), although heterogeneity was relatively low (*I*^2^ = 46.41%, *p* = 0.06; adjusted *I*^2^ = 18.49%, *p* = 0.28), indicating that most studies were in agreement.

### 3.4. Subgroup Analysis

For subgroup analysis concerning the primary outcome of locoregional recurrence, seven studies were identified in the radiation-plus-chemotherapy subgroup, and six in the radiation-only subgroup. Two of the radiation-only subgroup studies had local and distant recurrence combined into a single outcome [38,56]. Because the combined outcome is more stringent and has a tendency to favour the chemotherapy arm, it was reasonable to include these studies in the analysis. The study by Chen et al. (2011) is a prospective cohort study that compared radiotherapy against intravesical chemotherapy alone as adjuvant therapy for T3–4N± disease, and did not make use of any systemic chemotherapy, and so was included in the radiotherapy-alone subgroup.

**Figure 4:**
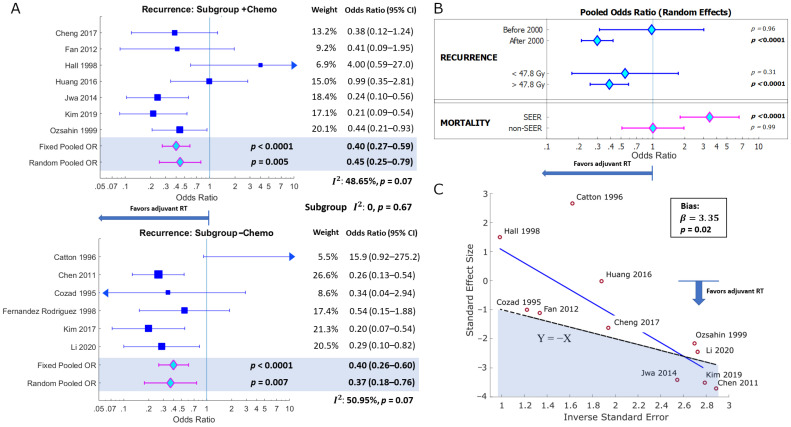
Subgroup analysis and small-study effects. (**A**) Forest plot for subgroup analysis of adjuvant radiotherapy with at least some patients receiving systemic chemotherapy (+Chemo) and those receiving radiotherapy alone (−Chemo). (**B**) Subgroup analyses of recurrence risk (top) for studies before or after the year 2000; (middle) studies delivering more or less than the median dose of 47.8 Gy for adjuvant RT, and (bottom) composite mortality (i.e., combining studies reporting 3- or 5-year OS) by SEER or non-SEER study subgroups. (**C**) Radial plot of results of Harbord’s Test. Studies with high variance are on the left-hand side of the plot. The bias term (*β*) refers to the Y-intercept of the regression line (solid blue). Unbiased studies should be distributed symmetrically about |Y| = |X| (segmented black line), but a small-study bias is detected.

Results of chemotherapy subgroup analysis are presented in Figure 4A. Pooled odds ratios were significant and favourable for both the radiotherapy-plus-chemotherapy (+*Chemo*) and radiotherapy-alone (−*Chemo*) subgroups (+*Chemo*: *ORp* = 0.45 (95% CI: 0.25–0.79), *p* = 0.005; −*Chemo*: *ORp* = 0.37 (95% CI: 0.18–0.76), *p* = 0.007). Within-subgroup study heterogeneity was moderate, but non-significant for the two subgroups (+*Chemo*: *I*^2^ = 48.65%, *p* = 0.07; −*Chemo*: *I*^2^ = 50.95%, *p* = 0.07), suggesting relative consistency in reported effect between studies within each subgroup. Between-subgroup heterogeneity was negligible (*I*^2^ = 0, *p* = 0.67), indicating no significant difference between subgroups, with both subgroups favouring adjuvant therapy. This result also supports the hypothesis that adjuvant radiotherapy alone has a significant effect on reducing locoregional recurrence.

Additional subgroup analyses of primary and secondary outcomes are shown in Figure 4B. Comparing studies published before and after the year 2000, older studies had higher variance and revealed no benefit to adjuvant RT in reducing recurrence risk (*ORp* = 0.97 (95% CI: 0.31–3.02), *p* = 0.96), whereas newer studies did demonstrate a robust benefit (*ORp* = 0.30 (95% CI: 0.21–0.42), *p* < 0.0001) (between subgroup heterogeneity *I*^2^ = 75.16%, *p* = 0.04), suggesting an advantage to more modern radiotherapy approaches. A dose–response with respect to reduced recurrence risk was also observed in subgroup analysis of studies that delivered adjuvant radiotherapy to a higher dose than the median of 47.8 Gy (*ORp* = 0.39 (95% CI: 0.26–0.59), *p* < 0.0001), compared with those that delivered a lower dose (*ORp* = 0.55 (95% CI: 0.17–1.74), *p* = 0.31) (between subgroups: *I*^2^ = 0, *p* = 0.59). Finally, because only a handful of studies reported on overall survival, including the SEER studies, which had an outsized effect on the analysis due to their large sample sizes, we compared SEER and non-SEER studies based on composite overall survival (which combined studies reporting 3- or 5-year OS). The subgroup analysis showed the SEER studies were the primary contributors to the apparent negative effect of adjuvant RT on survival (*ORp* = 3.43 (95% CI: 1.81–6.51), *p* < 0.0001), whereas the non-SEER studies did not demonstrate any survival impact, unfavourable or otherwise (*ORp* = 1.00 (95% CI: 0.51–1.96), *p* = 0.99) (between subgroups: *I*^2^ = 86.28%, *p* = 0.007).

### 3.5. Small-Study Effects

Results of Harbord’s test are summarized in the radial plot in Figure 4C. Studies with low variance (high inverse error) demonstrated low bias, being fairly symmetrically distributed across the *Y* = −*X* line. The negative values of their standard effect sizes indicate reduced locoregional recurrence rates with adjuvant radiotherapy. However, smaller studies and studies with higher variance (appearing on the left-hand side of the plot) had a tendency to cluster above the *Y* = −*X* line, suggesting a small-studies bias was present that was in the direction of the null or unfavourable effect of adjuvant radiotherapy on locoregional control (Harbord bias: *β* = 3.35, *p* = 0.02). The majority of these biased studies tended to be smaller in sample size and older (i.e., published prior to year the 2000) than their less-biased counterparts.

## 4. Discussion

Our systematic review and meta-analysis demonstrates that adjuvant radiotherapy significantly reduces odds of locoregional recurrence (by over one-half) in comparison to no adjuvant therapy, adjuvant chemotherapy or salvage radiotherapy. For most studies, adjuvant radiotherapy was administered for high-risk disease (i.e., non-organ confined, node positive and/or margin positive disease). The beneficial effect of radiation persisted even after subgroup analysis that accounted for adjuvant chemotherapy given either concomitantly or sequentially with radiation therapy. In regard to 3-year overall survival, 5-year cancer-specific survival and distant recurrence, adjuvant radiotherapy did not appear to have a significant effect on these outcomes. However, OS at 5 years was worse in the adjuvant radiotherapy arm, although study heterogeneity was high, indicating discordance in reported outcomes between studies.

Some of the publications we assessed included multivariable and propensity-score adjusted modeling of the effect of covariates on outcomes in addition to adjuvant radiotherapy. Overall, lower age, stage, grade and total nephrectomy with clear margins correlated with improved outcomes [14,29,30,33,48,50]. Adjuvant radiotherapy was associated with improved locoregional control on adjusted analysis [31,33,53,55], but survival results were not conclusive [14,30,31]. Smoking did not factor into our analysis as only one study reported on it (and multivariable analysis by the authors of that study did not show any statistical impact on outcomes), but independent of radiotherapy, smoking has been shown to be a negative prognostic factor in UTUC, and associated with worse outcomes following treatment [12,57]. Gender and ethnicity were not significant in terms of impact on outcomes [29,30], in keeping with the conflicting or non-conclusive results of studies which were not included within the scope of our review and meta-analysis [4,15,58].

The pooled outcomes for overall and cancer-specific survival were heavily influenced by a few studies. In particular, the two studies that analysed the role of radiotherapy from the SEER database, those by Hahn et al. (2016) and Ding et al. (2017), had disproportionately large sample sizes (*N* = 2572 and 1910, respectively), comprising 69% of the 6529 total patients across all included studies. These SEER-based studies also reported the lowest overall and cancer-specific survival rates for adjuvant radiotherapy out of any of the studies included (see Table 4). The remaining studies demonstrated non-significant or favourable effect of adjuvant radiotherapy on survival. One of the primary reasons for this, as made explicit by Hahn et al. (2016), was that the number of patients receiving adjuvant radiotherapy was very small in comparison to those that did not receive radiotherapy (4.4% vs. 95.6%), but the proportion of those with more advanced disease and/or disease with high-risk features was significantly higher in the adjuvant radiotherapy arm. Those with advanced, non-organ-confined disease comprised 31.4% vs. 6.2% of patients in the adjuvant RT vs. no adjuvant RT arms, respectively, and poorly differentiated histology was associated with 96.4% vs. 84.7% of patients in the respective arms, suggesting a strong selection bias. When these and other covariates were adjusted for in multivariate analysis, the effect of radiotherapy on overall survival became non-significant (hazard ratio = 0.68 (0.68–1.06), *p* = 0.85) [30]. Several other studies reached a similar conclusion regarding the use of post-operative radiotherapy [31,32,54]. Ding et al. (2017) reported a very small percentage of patients who received adjuvant radiotherapy (6.7%) but the actual distribution of early stage and locally advanced disease for adjuvant radiotherapy was unclear. Details of radiotherapy techniques and dose-fractionation were not reported in either SEER study, due to limitations of the data source. However, it is likely that the majority of radiotherapy patients had more advanced disease, and therefore survival would be expected to be worse. The authors of the study do admit their conclusions are weakened due to limitations of the SEER database in terms of data heterogeneity and confounding effects, whereby patients with more advanced disease and additional medical comorbidities were more likely to receive radiation and have a shorter survival time. Our own subgroup analysis supports the conclusion that there is probable bias in reporting of survival outcomes in the SEER studies compared to the non-SEER studies, of which the latter did not show a negative impact of adjuvant RT on survival (see Figure 4B).

Catton et al. (1996) and Hall et al. (1998), were the only studies to report worse locoregional control with adjuvant radiotherapy. These studies, unlike the SEER studies, had small sample sizes (*N* = 101 and 74, respectively), but likewise had imbalanced study arms. In the study by Catton et al., there were only 15 patients (14.8%) in the non-radiotherapy arm. Furthermore, there were no deaths or recurrences in that arm, which meant the odds ratio was undefined unless a continuity correction was applied. Similarly, Hall et al. only reported specific numbers for adjuvant radiotherapy versus no adjuvant therapy for Stage III patients, making sample size small (adjuvant RT, *N* = 15; no RT, *N* = 34), with very low numbers of locoregional recurrences in either arm (3 for adjuvant RT and 2 for no RT). As a consequence, these studies had odds ratios with disproportionately large effect size, which increased heterogeneity. Beyond heterogeneity however, these studies were also older (published before the year 2000) and implemented antiquated radiotherapy techniques. Both used a simple parallel-opposed pair for beam arrangement, and had heterogeneous prescription doses (dose range: 20–60 Gy for Catton et al. and 10–60 Gy for Hall et al.), with median total doses of 35 Gy and 39.8 Gy, respectively, which were much lower than the pooled study median of 47.8 Gy. Our subgroup analysis of adjuvant RT dose showed a dose–response relationship whereby the higher-dose subgroup (median dose > 47.8 Gy) had reduced recurrence risk, whereas the lower-dose subgroup did not. Thus, it is likely that target volumes were underdosed, which resulted in worse locoregional control, combined with larger, less conformal radiation fields that likely increased toxicity compared to contemporary studies utilizing more modern radiotherapy techniques such as 3D-CRT and IMRT.

The situation is similar when examining both primary and secondary outcomes in other non-SEER studies. Those that reported unfavourable outcomes for adjuvant radiotherapy (including single-arm studies not included in the meta-analysis) tended to be older studies, consistent with our own findings from subgroup analysis of articles published before and after the year 2000. Out of the eight non-SEER studies reporting one or more unfavourable outcomes for adjuvant radiotherapy, six were published before the year 2000. Indeed, the studies by Catton et al. and Hall et al., as well as the majority of older studies in our meta-analysis, accounted for much of the small-study bias that was statistically significant by Harbord’s Test, and directed toward the null or unfavourable effect of adjuvant radiotherapy when looking at the primary outcome of locoregional control. This suggests that overall, we can place less emphasis on these smaller, older studies in ascertaining the effect of adjuvant radiotherapy on outcomes.

Other limitations of our review are primarily related to the quality of studies used in the meta-analysis. There were no high-quality randomized controlled trials. All studies except for one were retrospective, and most were small studies with sample sizes below 200 patients. Therefore, study variance was high, with reasonable chance of confounding factors and bias in addition to what has already been discussed (see Table 3). Additionally, the majority of the studies published after the year 2000 originate from institutions in Asia, as adjuvant radiotherapy for locally advanced upper tract urothelial carcinoma is a more common practice there. This limits the generalizability of our study conclusions for patients outside of Asia. Furthermore, our study’s comparator arm was mixed, because different studies compared adjuvant radiotherapy to various treatment modalities including no further therapy, chemotherapy and/or salvage radiotherapy. Overall, there was insufficient quantity or quality of studies to perform subgroup analyses on each of these different comparator modalities. We also lacked the ability to perform granular analysis of the effect of adjuvant radiotherapy for different risk subgroups, such as those with positive surgical margins, or those with nodal disease. There is also the likelihood of some degree of overlap between patient populations between studies, in particular the SEER studies; however, although overlap could lead to overestimation of the sampling precision, it is in and of itself not a bias [59].

Our subgroup analysis of studies in which patients received adjuvant radiation alone, compared to those who received combined systemic chemotherapy and radiotherapy, showed that the improvement in locoregional control with radiation was present regardless of chemotherapy administration. However, we have not looked specifically in this study at the role of chemotherapy in improving survival or recurrence risk. A previous meta-analysis showed a trend toward improved overall survival with either adjuvant chemotherapy or neoadjuvant chemotherapy, with combined peri-operative pooled odds-ratio (OR) of 0.75 (95% CI: 0.57–0.99, *p* = 0.05) for overall survival; for adjuvant chemotherapy, a cancer-specific survival OR of 0.69 (95% CI: 0.42–1.15, *p* = 0.16) and disease-free survival OR of 0.54 (95% CI: 0.32–0.92, *p* = 0.02) were obtained [20]. This suggests that chemotherapy in addition to radiotherapy might yield better survival outcomes compared to radiotherapy alone, provided the combination does not increase toxicity significantly. However, the authors did not look into the effect of combining chemotherapy with radiotherapy. Similar benefit of adjuvant and neoadjuvant chemotherapy was reported in another earlier meta-analysis by Yang et al., with hazard ratios (HR) with respect to OS, CSS and recurrence-free survival (RFS) for adjuvant chemotherapy of 0.68 (95% CI: 0.51–0.89), 0.71 (95% CI: 0.54–0.89) and 0.49 (95% CI: 0.23–0.85), respectively; and for neoadjuvant chemotherapy, HRs of 0.46 (95% CI: 0.13–1.07), 0.25 (95% CI: 0.06–0.61) and 0.39 (95% CI: 0.02–1.33), for OS, CSS and RFS, respectively [21]. Interestingly, the effect of adjuvant RT in their analysis was not statistically significant in any category except for RFS when RT was combined with intravesical chemotherapy (HR 0.32, 95% CI: 0.03–0.97). Yang et al. report their results with respect to adjuvant RT contradict a number of other prior studies, and suggest their results were likely affected by the small sample sizes of radiotherapy patients and confounding factors such as different tumour grades and clinical stages which they were unable to adjust for.

## 5. Conclusions

The addition of adjuvant radiotherapy following definitive surgery (radical nephroureterectomy) for locally advanced upper urothelial tract carcinoma appears to result in improved locoregional control; however, overall survival appeared to be worse at 5 years and not significantly different at 3 years. Nevertheless, the conclusion that overall survival is negatively impacted by adjuvant radiotherapy is far from certain. Estimation of the effect of radiotherapy on OS and CSS is challenging because of small radiotherapy study arms, as well as selection bias in terms of patients who received RT having higher-risk disease and more comorbidities. Adjuvant RT was more likely to be given to sicker patients who underwent partial nephroureterectomies, and because of heterogeneity in treatment techniques, many older studies relied on antiquated treatment modalities that likely underdosed target volumes and increased toxicity through use of larger, less conformal radiotherapy fields, leading to poorer outcomes than expected. Therefore, our results are hypothesis-generating, given the lack of prospective studies with adequate power to investigate the role of radiation treatment in the adjuvant setting. Randomized controlled trials (RTCs) utilizing modern radiotherapy techniques remains the gold standard, but RTCs would be difficult to accrue because of the rarity of UTUC. Nevertheless, the suggested survival benefit from adjuvant and neoadjuvant chemotherapy, and more recently, immunotherapy [60], indicates that the combination of systemic therapy with radiotherapy might provide a complementary survival benefit to the locoregional control benefit of RT, possibly making such combined-modality treatments a sensible option in the setting of locally advanced or margin-positive UTUC, similar to what has been the trend for other genitourinary malignancies [61]. More evidence is needed, however, before this treatment approach can be recommended as a standard of care.

## Figures and Tables

**Table 1:** Study inclusion and exclusion criteria.

Inclusion Criteria	Exclusion Criteria
1. Patients with transitional-cell carcinoma (TCC) histology 2. Proximal ureter or renal pelvis location 3. Locally advanced disease at time of diagnosis (T3, T4 or node positive disease) 4. Adjuvant external beam radiotherapy 5. RCTs, prospective and retrospective cohort studies, or case series 6. Quantitative survival measures including but not limited to overall survival, cancer-specific survival, progression-free survival, locoregional recurrence rate, distant metastasis-free survival	1. Exclusively or primarily non-TCC histology 2. Distal ureter or bladder location only 3. Patients with distant metastases at the time of radiotherapy 4. No adjuvant radiotherapy (e.g., only salvage or palliative RT) 5. Stereotactic body radiotherapy 6. Brachytherapy 7. Intraoperative Radiotherapy 8. Subjective and qualitative outcome measures only (e.g., patient surveys) 9. Single case reports, review articles, expert guidelines, opinion articles, editorials, book chapters 10. Pediatric patients 11. Studies older than 1990

**Table 2:** Baseline Characteristics.

First Author	Year	Origin	Study Type	No.	Age (m)	M:F	No. Adj RT	No. Chemo	Surgery		T1-2	T3-4	N+	N0/Nx	Margin +	RT Type	RT Volume	D[Gy]/F (m)
									RNU	Partial								
Catton [52]	1996	Canada	RC	101	62	2	86	0	54	47	-	-	41	24	-	POP	TB + RLN	35/20
Chen [48]	2011	China	PC	133	68	1.66	67	66 (I)	111	22	81	52	9	124	25	3DC	TB + RLN	50/25
Cheng [53]	2017	Taiwan	RC(A)	106	-	-	20	-	106	-	-	-	-	-	-	-	-	50.4/28
Cozad [54]	1995	USA	RC	67	65	-	10	2	54	13	42	25	3	64	6	3DC	TB +/− margin	48/-
Czito [39]	2004	USA	CS	31	67	0.94	31	9	25	6	5	26	10	4	10	-	TB + RLN	46.9/-
Ding [29]	2017	China	SEER	1910	74	1.40	146	0	-		1174	706	210	1700	-	-	-	-
Fan [55]	2012	Taiwan	RC	40	61	0.90	20	34	40	0	0	20	5	4	3	3DC	TB + P.A. LN	50/25
Fernandez Rodriguez [38]	1998	Spain	RC	51	63.8	-	16	0	32	19	28	23	-	-	-	3DC	Renal fossa + RLN	55/-
Hahn [30]	2016	USA	SEER	2572	74	1.32	113	-	1084	1488	L (780)	----NL---- (1792)		-	-	-	-	-
Hall [49]	1998	USA	RC	74	67.5	1.85	28	10	64	10	179	65	15	229	-	POP +/− lateral boost	TB + RLN	39.8/-
Huang [51]	2016	Taiwan	RC	198	68.6	0.92	40	21	198	0	0	198	0	198	9	3DC/IMRT	TB + RLN	50.4/28
Jwa [31]	2014	Korea	RC	127	64	1.89	36	47	127	-	2	125	18	37	20	3DC	TB + RLN	46/23
Kang [34]	2020	Taiwan	CS	21	65.5	1.62	16	21	-	-	-	-	8	13	7	-	TB + RLN	
Kim [32]	2019	Korea/Japan	RC	222	68	1.92	39	74	222	0	0	222 (T3b)	-	222	24	3DC	TB	46/23
Kim [56]	2017	Korea	RC(A)	128	-	-	41	-	-	-	0	128 (T3b)	-	128	-	-	-	-
Li [33]	2020	China	RC	389	69	0.95	57	0	389	0	246	138	30	43	12	VMAT	TB + RLN	50/25
Matsuoka [9]	1991	Japan	RC	100	64	2.85	24	85	75	15	51	56	-		-	-	-	-
Maulard-Durdux [40]	1996	France	CS	26	65	-	26	4	22	4	11	15	9	12	-	POP	TB +/− RLN	45/35
Ozsahin [50]	1999	Europe (8 centers)	RC	126	66	2.50	45	10	111	15	39	74	26	69	33	POP	Renal fossa + ureteral bed +/− bladder	50/25
Saito [37]	2009	Japan	RC	107	67	3.86	18	18	100	7	56	47	15	92	-	-	TB + RLN	46.9/21
**Total/[W]**	-	-	-	6529	[71.9]	[1.4]	879	401	2814	1646	1914	1920	399	2963	149	-	-	[47.8/-]

3DC—3D conformal; A—abstract; Adj—adjuvant; CS—case series; D—dose; F—fractions; Gy—Gray; I—Intravesical; IMRT—intensity modulated radiation therapy; L—localized; M:F—male-to-female ratio; NL—non-localized; N—node; No.—Number; m— median; PC—prospective cohort; POP—parallel opposed pair; RC—retrospective cohort; RLN—regional lymph nodes; RNU—radical nephroureterectomy; RT—radiotherapy; SEER—Surveillance, Epidemiology and End Results, T—tumor (stage); TB—tumor bed; VMAT—volumetric modulated arc therapy; W—weighted average.

**Table 3:** Study quality (MINORS criteria).

Study Type		Quality		
Comparative Studies		Lower	Intermediate	Higher
		1–12	13–17	18–24
Catton [52]	1996	10		
Chen [48]	2011			19
Cheng [53]	2017		13	
Cozad [54]	1995	10		
Ding [29]	2017		15	
Fan [55]	2012		16	
F-Rodriguez [38]	1998	11		
Hahn [30]	2016		16	
Hall [49]	1998		13	
Huang [51]	2016		16	
Jwa [31]	2014			18
Kim [32]	2019		15	
Kim [56]	2017	10		
Li [33]	2020		15	
Matsuoka [9]	1991	8		
Ozsahin [50]	1999		13	
Saito [37]	2009	9		
**Case series**		**Lower**	**Higher**	
		1–8	9–16	
Czito [39]	2004		9	
Kang [34]	2020	8		
M-Durdux [40]	1996	7		

Note: shaded bars indicate quality category corresponding to each study score

**Table 4:** Study outcomes.

Study		Overall Survival						Cancer Specific Survival (%)				Progression-Free Survival				Recurrence (%)			
		2 or 3-yr (%)		5-yr (%)		Median (mo)		2 or 3-yr (%)		5-yr (%)		2 or 3-yr (%)		Median (mo)		Locoregional		Distant	
		aRT	No aRT	aRT	No aRT	aRT	No aRT	aRT	No aRT	aRT	No aRT	aRT	No aRT	aRT	No aRT	aRT	No aRT	aRT	No aRT
Catton [52]	1996									43.0	100.0					33.7	0	47.7	0
Chen [48]	2011	61.1	53.6	49.6	44.7	55.0	52.4									31.3	63.6	23.9	22.7
Cheng [53]	2017							84.4	73.1							18.5	39.1	26.9	40.9
Cozad [54]	1995															10.0	24.6	55.6	56.3
Czito [39] ‡	2004			39	⸻	28.8	⸻			52	⸻					23	⸻	48	⸻
Ding [29]	2017			15.6	47.2	21	55			22.7	60.3								
Fan [55]	2012	45.0	16.0			29	15					41	12	21	6	15.0	30.0	45.0	40.0
F-Rodriguez [38]	1998															[⸻	47	31	⸻]
Hahn [30]	2016	31	46	24	44	19	31												
Hall [49]	1998					7 **	9 **			45 *	40 *					20	6		
Huang [51]	2016	73.4 ^#^	72.0 ^#^			29.6	29	75.3 ^#^	73.2 ^#^			66.3 ^# $^	61.2 ^# $^			12.5	8.2	32.2	25.0
Jwa [31]	2014	66	62													27.8^	61.5^	41.6	46.2
Kang [34] ‡	2020	57.6	⸻			37.6	⸻					21.1	⸻						
Kim [32]	2019									76.4	55.5					16.1	45.8	27.9	51.9
Kim [56]	2017									61	51					[⸻	70.1	37.6	⸻]
Li [33]	2020															7.0	20.8		
Matsuoka [9]	1991			41.5	63.1														
M-Durdux [40] ‡	1996			49	⸻											19.2	⸻	53.8	⸻
Ozsahin [50]	1999			20	33											37.8	58.0	33.3	24.7
Saito [37]	2009									73	78								

aRT—adjuvant radiotherapy; mo—months; yr—year; ‡ case series; * stage III patients only; ** stage IV patients only; ^#^ 2-year survival; ^$^ recurrence-free survival; ^ includes bladder recurrences; [⸻ ⸻] includes both locoregional and distant recurrences
